# Female Sex and Mortality in Patients with *Staphylococcus aureus* Bacteremia: A Systematic Review and Meta-analysis

**DOI:** 10.1001/jamanetworkopen.2024.0473

**Published:** 2024-02-27

**Authors:** Annette C. Westgeest, Merel M. C. Lambregts, Felicia Ruffin, Rachel E. Korn, Maren E. Webster, Jackson L. Kair, Joshua B. Parsons, Stacey A. Maskarinec, Samantha Kaplan, Olaf M. Dekkers, Mark G. J. de Boer, Vance G. Fowler, Joshua T. Thaden

**Affiliations:** 1 Division of Infectious Diseases, Duke University, Durham, North Carolina; 2 Department of Infectious Diseases, Leiden University Medical Center, Leiden, the Netherlands; 3 Medical Center Library and Archives, Duke University, Durham, North Carolina; 4 Department of Clinical Epidemiology, Leiden University Medical Center, Leiden, the Netherlands; 5 Duke Clinical Research Institute, Durham, North Carolina

## Abstract

**Importance:**

*Staphylococcus aureus* is the leading cause of death due to bacterial bloodstream infection. Female sex has been identified as a risk factor for mortality in *S aureus* bacteremia (SAB) in some studies, but not in others.

**Objective:**

To determine whether female sex is associated with increased mortality risk in SAB.

**Data Sources:**

MEDLINE, Embase, and Web of Science were searched from inception to April 26, 2023.

**Study Selection:**

Included studies met the following criteria: (1) randomized or observational studies evaluating adults with SAB, (2) included 200 or more patients, (3) reported mortality at or before 90 days following SAB, and (4) reported mortality stratified by sex. Studies on specific subpopulations (eg, dialysis, intensive care units, cancer patients) and studies that included patients with bacteremia by various microorganisms that did not report SAB-specific data were excluded.

**Data Extraction and Synthesis:**

Data extraction and quality assessment were performed by 1 reviewer and verified by a second reviewer. Risk of bias and quality were assessed with the Newcastle-Ottawa Quality Assessment Scale. Mortality data were combined as odds ratios (ORs).

**Main Outcome and Measures:**

Mortality at or before 90-day following SAB, stratified by sex.

**Results:**

From 5339 studies retrieved, 89 were included (132 582 patients; 50 258 female [37.9%], 82 324 male [62.1%]). Unadjusted mortality data were available from 81 studies (109 828 patients) and showed increased mortality in female patients compared with male patients (pooled OR, 1.12; 95% CI, 1.06-1.18). Adjusted mortality data accounting for additional patient characteristics and treatment variables were available from 32 studies (95 469 patients) and revealed a similarly increased mortality risk in female relative to male patients (pooled adjusted OR, 1.18; 95% CI, 1.11-1.27). No evidence of publication bias was encountered.

**Conclusions and Relevance:**

In this systematic review and meta-analysis, female patients with SAB had higher mortality risk than males in both unadjusted and adjusted analyses. Further research is needed to study the potential underlying mechanisms.

## Key Points

**Question:**

Is female sex associated with increased mortality risk in patients with *Staphylococcus aureus* bacteremia?

**Findings:**

In this systematic review and meta-analysis that included 132 582 patients from 89 studies, female sex was associated with increased mortality. Female patients with *S aureus* bacteremia had 18% increased odds of death compared with male patients.

**Meaning:**

These results suggest that female patients with *S aureus* bacteremia have higher mortality risk than male patients; further research is needed to study the potential underlying mechanisms.

## Teaser

This systematic review and meta-analysis of studies on outcomes for patients hospitalized with *Staphylococcus aureus* examines the association of mortality with female sex.

## Introduction

*Staphylococcus aureus* is the leading cause of death due to bacterial bloodstream infection.^1^ Previously identified risk factors for mortality in patients with *Staphylococcus aureus* bacteremia (SAB) have included increasing age, infective endocarditis, hemodialysis dependence, and persistent bacteremia, among others.^2^ Female sex has been suggested as risk factor for mortality in SAB in several studies, with an increase of mortality of up to 30% relative to male patients.^3,4,5^ However, other studies found no gender inequality in outcome of SAB,^6,7^ or even a higher mortality in male individuals in a subgroup of patients with a higher comorbidity score.^8^ Thus, the impact of female sex in SAB remains unclear. The aim of this systematic review and meta-analysis was to determine whether female sex is associated with mortality in SAB.

## Methods

The key question of this systematic review was: is female sex associated with increased mortality risk in patients with SAB? The study protocol was registered on Prospero (CRD42022373176). We followed the meta-analysis of observational studies in epidemiology Meta-analysis of Observational Studies in Epidemiology (MOOSE) reporting guideline as the included studies involved observational data.

### Search Strategy

We conducted a literature search of MEDLINE via PubMed, Embase via Elsevier, and Web of Science Core Collection (1900 to present) via Clarivate from inception to October 31, 2022, using a combination of key words to capture *S aureus,* bacteremia, mortality, and sex (eAppendix 1 in Supplement 1). An experienced medical librarian (S.K.) devised, developed, and executed the search with input from the entire team. The search was peer reviewed by a second medical librarian according to a modified Peer Review of Electronic Search Strategies (PRESS) checklist.^9^ No limitations were placed on language in the initial search, but studies published in languages other than English were excluded in the full-text review phase. A search update was conducted on April 26, 2023, to identify newly published studies. In addition, we hand-searched key references to identify citations not captured in the electronic database searches. All results were compiled in EndNote and imported into Covidence, a web-based data synthesis software program,^10^ for deduplication and screening.

### Study Selection, Data Extraction, and Quality Assessment

We included studies that met the following conditions: (1) randomized or observational study evaluating outcomes in adults with SAB, (2) included 200 or more patients, (3) reported mortality at or before 90 days following SAB, and (4) reported mortality stratified by sex. Exclusion criteria were studies on specific subpopulations (eg, dialysis, intensive care unit, hematological or oncological patients), studies that included SAB patients as a subgroup (eg, patients with bacteremia by any microorganism) that did not report SAB-specific data, and studies using (partially) the same cohort as another study included in this review. In this latter scenario, the study with the largest cohort was included. Titles and abstracts of articles (with authors and institutions visible) identified through our primary search were screened independently by 2 reviewers (A.W. reviewed all; R.K., M.W., J.K., F.R., J.P., S.M., S.K., M.L., V.F., and J.T. were second reviewers). Conflicts at this stage were resolved by a third person. Articles marked for full-text review underwent full-text screening by 2 independent reviewers. Conflicts at this stage were resolved by consensus or by obtaining a third reviewer’s opinion when consensus could not be reached. Data extraction and quality assessment was done by 1 reviewer and verified by a second reviewer. Extracted variables included lead author, journal, year of publication, start and end year of inclusion, country, aim of study, study design, number of hospitals, number of patients, population description, and whether methicillin-resistant *S aureus* (MRSA), methicillin-susceptible *S aureus* (MSSA), or both were addressed. Unadjusted mortality stratified by sex was extracted, as well as adjusted mortality when reported, the statistical model and the covariates for which mortality was adjusted. If a study described mortality for 2 subgroups (eg, for MSSA and MRSA bacteremia separately), both were included. Risk of bias and quality were assessed with the Newcastle-Ottawa Quality Assessment Scale^11^ (eAppendix 2 in Supplement 1) because only observational studies were identified.

### Statistical Analysis

Mortality data were combined as odds ratios (ORs). If ORs were not reported in a study, we calculated ORs from raw mortality by sex if such data was available. If raw data was not available either, then ORs were calculated from the provided risk ratio (RR) or hazard ratio (HR) values based on previously published methods.^12,13^ In the single study that reported a rate ratio,^14^ this rate ratio was used to estimate the OR.^15^ Sensitivity analyses involving only studies that directly reported an OR (as opposed to estimating OR based on HR or RR) were conducted. ORs were combined using inverse variance with random effects models. We used the Knapp and Hartung method to adjust the standard errors of the estimated coefficients.^16,17^ Robustness of findings were assessed through influence and sensitivity analyses as detailed in the text. We evaluated statistical heterogeneity with the Cochran *Q* and *I^2^* statistics. To explore potential sources of heterogeneity, we performed meta-analyses on subsets of studies to determine if variation in factors such as mortality time point (eg, 30-day vs 90-day mortality), bacterial groups (eg, MSSA only, MRSA only, both MSSA and MRSA), or geographic location between studies could be contributing. Statistical analyses were performed with RStudio version 2022.02.0 (R Project for Statistical Computing). Publication bias was assessed using funnel plots with the Egger test^18^ when 10 or more studies were included in the analysis. We used the Evidence-based Practice Center (EPC) model from the US Agency for Healthcare Research and Quality (AHRQ) to grade overall strength of evidence.^19^ A full description of the EPC approach is detailed in eAppendix 3 in Supplement 1.

## Results

**Figure 1.**
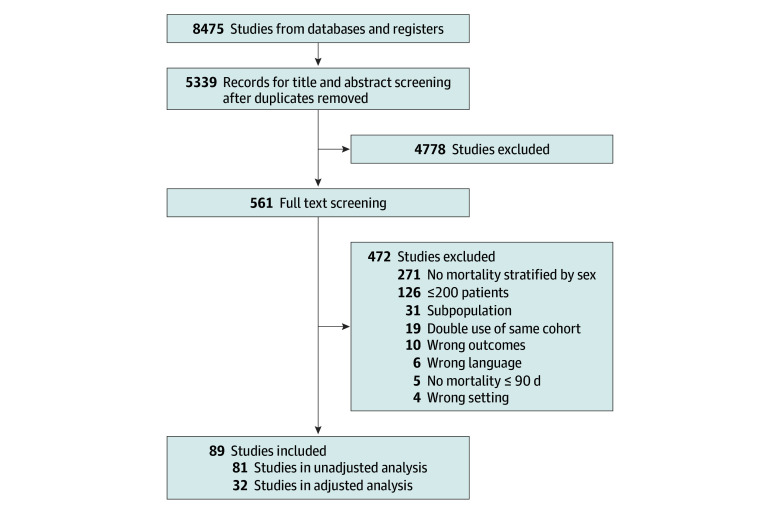
Search Flow Diagram of Systematic Review

We screened the title and abstract of 5339 studies, and 4778 were deemed irrelevant (Figure 1). A full-text assessment was performed on 561 studies, and 472 of these were excluded. We included 89 studies in the analysis, with a total of 132 582 patients (50 258 female [37.9%], 82 324 male [62.1%]) (Table).^3,4,5,6,7,8,14,20,21,22,23,24,25,26,27,28,29,30,31,32,33,34,35,36,37,38,39,40,41,42,43,44,45,46,47,48,49,50,51,52,53,54,55,56,57,58,59,60,61,62,63,64,65,66,67,68,69,70,71,72,73,74,75,76,77,78,79,80,81,82,83,84,85,86,87,88,89,90,91,92,93,94,95,96,97,98,99,100,101^ All data on mortality by sex were from observational studies: 88 of 89 cohort studies and 1 post hoc analysis of a randomized clinical trial. Mortality was most frequently assessed at 28 to 30 days (54 of 89 studies [61%]). The majority of studies were conducted in Europe (36 [40%]), Asia (24 [28%]) and North America (20 [22%]). The majority of studies were published after 2010 (68 [76%]). Thirty-two studies (36%) were rated as having low risk of bias, and 57 studies (64%) as having high risk of bias (detailed quality assessment of each study in eTable 1 in Supplement 1).

**Table.** Description of Studies Included in Systematic Review

Study characteristics	Studies, No. (%) (N = 89)
Publication year	
2000-2010	21 (24)
2011-2023	68 (76)
Study design	
Cohort study	88 (99)
Post hoc analysis randomized trial	1 (1)
Continent	
Europe	36 (40)
Asia	24 (27)
North America	20 (22)
Oceania	5 (6)
South America	1 (1)
Africa	1 (1)
Multiple	2 (2)
No. of hospitals included	
1	44 (49)
2-20	33 (37)
>20	13 (15)
No. of patients included	
200-1000	69 (78)
1000-10 000	15 (17)
>10 000	4 (4)
Population	
All SAB patients	82 (92)
Health care/hospital-associated SAB	3 (3)
Community-acquired SAB	4 (4)
Outcome measure	
7-d mortality	1 (1)
14-d mortality	4 (4)
28-30–d mortality	54 (61)
90-d mortality	9 (10)
In-hospital mortality	16 (18)
Attributable mortality	5 (6)
MRSA vs MSSA	
Both MRSA and MSSA	59 (66)
Only MRSA	20 (22)
Only MSSA	10 (11)

Abbreviations: MRSA, methicillin-resistant *Staphylococcus aureus*; MSSA, methicillin-susceptible *Staphylococcus aureus*; SAB, *Staphylococcus aureus* bacteremia.

### Mortality by Sex

**Figure 2.**
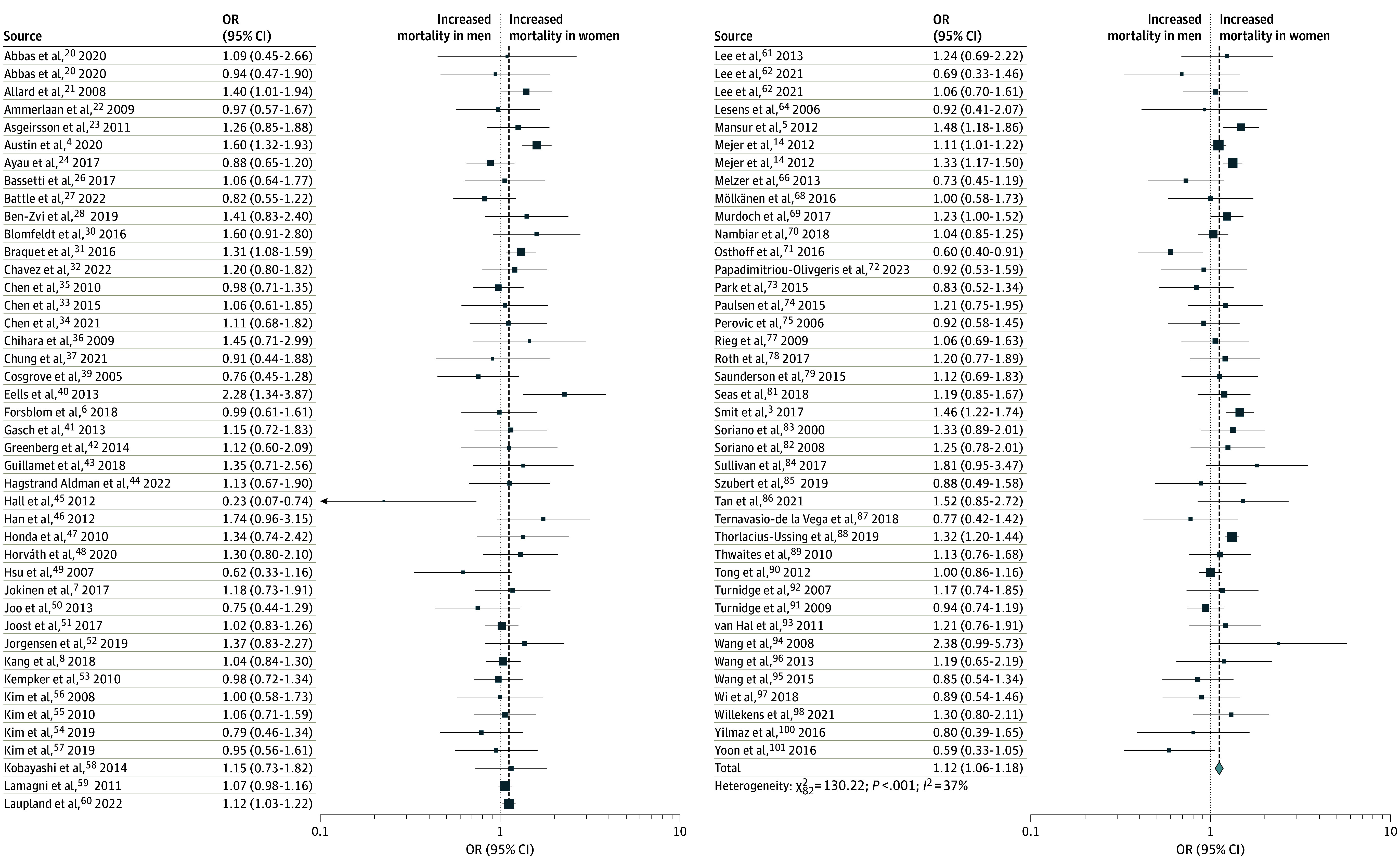
Forest Plot of Unadjusted Mortality in Female vs Male Patients With *Staphylococcus aureus* Bacteremia

Unadjusted mortality data was available from 81 studies (109 828 patients) and revealed an increased mortality risk in female compared with male patients (pooled OR, 1.12; 95% CI, 1.06-1.18) (Figure 2). Moderate heterogeneity was observed in this analysis (Q = 130.17; *P* < .001; *I*^2^ = 37%). An influence analysis revealed that exclusion of any single study did not significantly alter the findings from the overall cohort (eAppendix 4 in Supplement 1). A sensitivity analysis with only studies that had an OR that was either reported or could be directly calculated (ie, excluding 14 studies in which RR or HR were reported) similarly did not change the overall findings (eFigure 1 in Supplement 1). Exclusion of single-center studies did not change the overall findings. No funnel plot asymmetry was found (eFigure 2 in Supplement 1).

**Figure 3.**
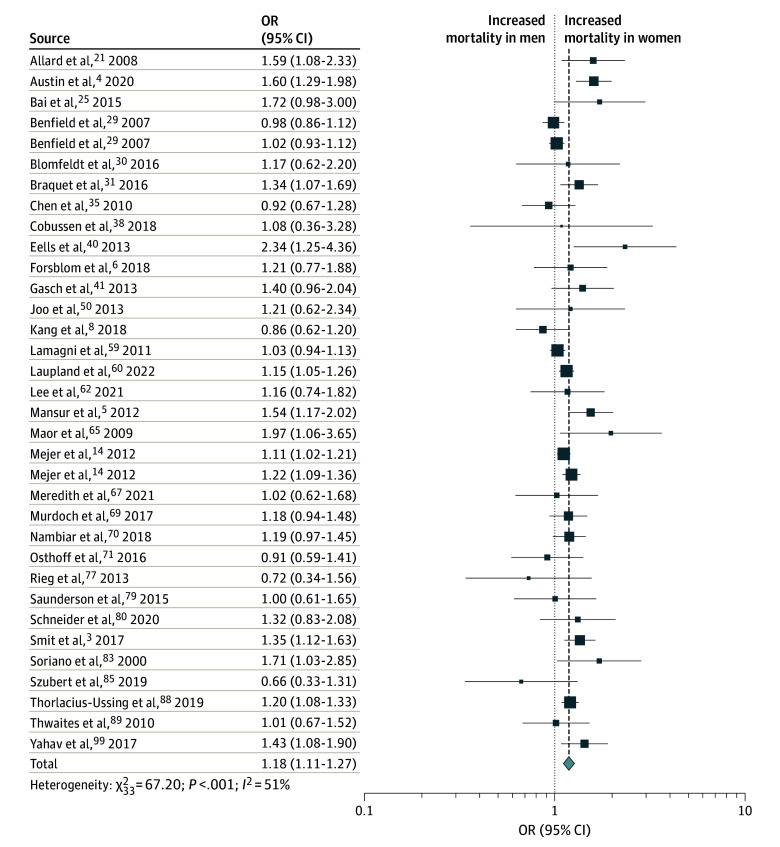
Forest Plot of Adjusted Mortality in Female vs Male Patients With *Staphylococcus aureus* Bacteremia

Adjusted mortality data that accounted for patient characteristics and treatment variables was available from 32 studies (95 469 patients) and revealed a similarly increased mortality risk in female relative to male patients (pooled adjusted OR [aOR], 1.18; 95% CI, 1.11-1.27) (Figure 3). An influence analysis revealed that exclusion of any single study did not significantly alter the findings from the overall cohort (eAppendix 5 in Supplement 1). A sensitivity analysis with only studies that had an OR that was either reported or could be directly calculated (ie, excluding 14 studies in which RR or HR were reported) similarly did not change the overall findings (eFigure 3 in Supplement 1). No funnel plot asymmetry was found (eFigure 4 in Supplement 1). Substantial heterogeneity was observed in this analysis of adjusted mortality data (Q = 66.98; *P* < .001; *I*^2^ = 51%). Meta-analyses on subsets of studies showed that variation in the geographic location of the study impacted heterogeneity. Meta-analyses of studies conducted in individual geographic regions all had lower observed heterogeneity than the overall cohort (overall *I*^2^ = 51%): Europe (19 studies; *I*^2^ = 41%), North America (5 studies; *I*^2^ = 12%), East Asia (4 studies; *I*^2^ = 0%), and Middle East (3 studies; *I*^2^ = 0%). The pooled aOR varied significantly based on geographic location of study and ranged from 0.96 (95% CI, 0.76-1.22) for studies conducted in East Asia to 1.57 (95% CI, 1.23-2.01) for studies conducted in North America. Stratification of studies by mortality time point or by methicillin resistance did not impact heterogeneity.

### Evaluation of the Evidence

Given that this systematic review contained observational studies that accounted for confounding through statistical adjustment (ie, the adjusted analysis), the baseline strength of evidence was moderate. The mortality effect estimate was downrated due to a serious risk of bias because studies without a sex-difference in a univariable analysis would likely not have included this variable in a multivariable analysis. We did not have serious concerns about inconsistency, indirectness, imprecision, or publication bias. Therefore, the overall strength of evidence for the association of female sex with increased mortality risk in patients with SAB was low (eTable 2 in Supplement 1).

## Discussion

In this systematic review and meta-analysis, we addressed the question of whether female sex is associated with increased mortality risk in patients with SAB. The included studies involved over 130 000 patients and identified an association between female sex and increased mortality risk in both unadjusted and adjusted analyses. Heterogeneity was observed, but substantially decreased with stratification by geographic region. This may reflect the large practice variations for SAB throughout the world, as recently described in a global survey.^102^

This study sheds new light on sex differences in clinical outcomes of patients with SAB, which is an area of little clarity. Few studies have primarily focused on sex differences in outcome in SAB patients, and their results have been contradictory. Some studies reported higher mortality in female patients with SAB compared with male patients,^3,5^ while others did not report an overall sex-difference in mortality.^6,8^ In this meta-analysis we identified a relatively large (18%) increased odds of death in female patients compared with male patients. This association was significant in both the unadjusted analysis and in an adjusted analysis that accounted for patient co-morbidities and treatment variables. Beyond patients with SAB, excess mortality has been reported in female patients with hospital-acquired bloodstream infection,^103^ severe sepsis,^104,105,106^ and endocarditis^107^; however, conflicting evidence has been reported as well.^108^

The underlying causes of sex differences in clinical outcomes of patients with SAB were not addressed in this study. Sex-related differences in outcome may be due to a variety of social or biological factors. Firm data for a biological connection between sex differences in clinical outcomes from animal models has been elusive. Previous studies on sepsis have generally supported better outcomes in female patients relative to male.^109^ This has been hypothesized to stem from the positive immunomodulatory properties of sex hormones on cell-mediated immune responses and cardiovascular functions in female patients^110,111^ as well as the suppression of the anti-infective response by testosterone in male patients.^112^ Even an ongoing immunological advantage in postmenopausal septic women has been reported.^113^ In *S aureus* infections in particular, an animal study showed enhanced neutrophil bactericidal capacity in female mice.^114^ However, females were more susceptible to lethal toxic shock caused by *S aureus* enterotoxin B in another mouse model.^115^ Social factors could also be contributing to the observed differences in mortality between female and male patients with SAB. Analogous to acute myocardial infarction, where women waited longer before seeking treatment relative to men, gender-differences in health seeking behavior may exist in SAB patients.^116^ Gender bias in health care delivery can potentially contribute to the difference in outcome as well. Delays in antibiotic treatment and less invasive treatment have been reported in women with septic shock and critical illness,^105,117,118,119^ and women were less likely to receive the recommended quality of acute care compared with men in a US study on quality of care in sociodemographic subgroups.^120^ In a 2023 cohort study from our research group,^121^ women with SAB received shorter durations of antimicrobial treatment and were less likely to undergo transesophageal echocardiography compared to men. Regional or cultural differences in health care delivery could be impacting the observed sex-based difference in patient outcomes. The association between female sex and mortality varied to some degree by location of study, and we have previously shown that there is considerable global variation in SAB treatment factors.^102^ Finally, response to treatment can differ between female and male patients. Both pharmacokinetics and pharmacodynamics are generally subject to sex influences.^122^

### Limitations

This study had several limitations. First, sex difference was not the primary outcome of interest in the majority of the included studies. Therefore, a number of studies did not include adjusted data for mortality by sex, and inclusion of this data could have influenced the results. Second, reporting bias can exist as studies may not report mortality stratified by sex if there was no significant difference in mortality. Third, heterogeneity exists not only in study methodology but also in the disease itself. The clinical presentation of SAB may vary from uncomplicated intravenous catheter-related bacteremia to complicated metastatic disease. Because all studies on SAB patients were included in our study, sex-based differences in outcome could not be stratified by infection severity. Lastly, whether reported sex represented sex assigned at birth or gender, was often not specified.

## Conclusions

In this systematic review and meta-analysis, observational cohort studies demonstrated an association between female sex and increased mortality risk in adult patients with SAB. This association remained significant after including only studies that adjusted for patient clinical and treatment variables. Future research should focus on understanding the underlying causes and on promoting better outcomes in female patients with SAB. Fundamental research on biological sex differences in immune response or pharmacology, examinations of sex-based differences in management of SAB, and better reporting of sex-specific outcomes in randomized clinical trials are necessary to better understand the observed sex-specific differences in mortality among patients with SAB.

## Notes

Supplement 1. **eAppendix 1.** Search Strategies **eAppendix 2.** Newcastle-Ottawa Quality Assessment Scale for Assessing Risk of Bias in Observational Studies **eAppendix 3.** Description of EPC Approach **eAppendix 4.** Influence Analysis Unadjusted Mortality **eAppendix 5.** Influence Analysis Adjusted Mortality **eTable 1.** Newcastle-Ottawa Quality Assessment of Individual Studies **eFigure 1.** Sensitivity Analysis Unadjusted Mortality **eFigure 2.** Funnel Plot Unadjusted Mortality **eFigure 3.** Sensitivity Analysis Adjusted Mortality **eFigure 4.** Funnel Plot Adjusted Mortality **eTable 2.** Evidence Profile for Association of Female Sex and Mortality in Patients With Staphylococcus Aureus Bacteremia

Supplement 2.
Data Sharing Statement
