# Supplementary material for: Female Sex and Mortality in Patients with Staphylococcus aureus Bacteremia: A Systematic Review and Meta-analysis
Source: JAMA Netw Open. 2024 Feb 27;7(2):e240473. doi: 10.1001/jamanetworkopen.2024.0473 (PMC10900971; doi:10.1001/jamanetworkopen.2024.0473)
Supplement: Supplement 1. — eAppendix 1. Search Strategies eAppendix 2. Newcastle-Ottawa Quality Assessment Scale for Assessing Risk of Bias in Observational Studies eAppendix 3. Description of EPC Approach eAppendix 4. Influence Analysis Unadjusted Mortality eAppendix 5. Influence Analysis Adjusted Mortality eTable 1. Newcastle-Ottawa Quality Assessment of Individual Studies eFigure 1. Sensitivity Analysis Unadjusted Mortality eFigure 2. Funnel Plot Unadjusted Mortality eFigure 3. Sensitivity Analysis Adjusted Mortality eFigure 4. Funnel Plot Adjusted Mortality eTable 2. Evidence Profile for Association of Female Sex and Mortality in Patients With Staphylococcus Aureus Bacteremia [file jamanetwopen-e240473-s001.pdf]

Supplemental Online Content

Westgeest AC, Lambregts MC, Ruffin F, et al. Female sex and mortality in patients with *staphylococcus aureus* bacteremia. *JAMA Netw Open.* 2024;7(2):e240473. doi:10.1001/jamanetworkopen.2024.0473

eAppendix 1. Search Strategies

eAppendix 2. Newcastle-Ottawa Quality Assessment Scale for Assessing Risk of Bias in Observational Studies

eAppendix 3. Description of EPC Approach

eAppendix 4. Influence Analysis Unadjusted Mortality

eAppendix 5. Influence Analysis Adjusted Mortality

eTable 1. Newcastle-Ottawa Quality Assessment of Individual Studies

eFigure 1. Sensitivity Analysis Unadjusted Mortality

eFigure 2. Funnel Plot Unadjusted Mortality

eFigure 3. Sensitivity Analysis Adjusted Mortality

eFigure 4. Funnel Plot Adjusted Mortality

eTable 2. Evidence Profile for Association of Female Sex and Mortality in Patients With Staphylococcus Aureus Bacteremia

This supplemental material has been provided by the authors to give readers additional information about their work.

© 2024 Westgeest AC et al. *JAMA Network Open.*

eAppendix 1. Search Strategy Report: Original Search

Topic: Association of female sex with mortality in patients with *Staphylococcus aureus* bloodstream infections

Searcher: SJK

Date: 10.31.2022 Updated 4.26.2023

Database (including vendor/platform): MEDLINE (via PubMed)

Set #	Search Strategy	Results
**#1 Staph**	"Staphylococcus aureus"[Mesh] OR "staphylococcus aureus"[tiab] OR "s. aureus"[tiab] OR "s aureus"[tiab] OR "staph aureus"[tiab]	**150579**
**#2 Infection**	"Endocarditis, Bacterial"[Mesh] OR "Bacteremia"[Mesh] OR bacteremia[tiab] OR bacteraemia[tiab] OR bacteremias[tiab] OR bacteraemias[tiab] OR bacteremic[tiab] OR bacteraemic[tiab] OR ((bloodstream[tiab] OR "blood stream"[tiab] OR bloodstreams[tiab] OR "blood streams"[tiab]) AND (infection[tiab] OR infections[tiab] OR infected[tiab] OR infect[tiab] OR infects[tiab] OR infecting[tiab])) OR endocarditis[tiab]	**105361**
**#3 Mortality**	"Mortality"[sh] OR "Mortality"[Mesh] OR mortality[tiab] OR mortalities[tiab] OR fatal[tiab] OR fatality[tiab] OR fatalities[tiab] OR death[tiab] OR deaths[tiab] OR dying[tiab] OR die[tiab] OR died[tiab]	**2467469**
**#4 Sex**	"Female"[Mesh] OR "Male"[Mesh] OR "Sex Factors"[Mesh] OR female[tiab] OR females[tiab] OR male[tiab] OR males[tiab] OR women[tiab] OR woman[tiab] OR "womens" OR "womans" OR men[tiab] OR gender[tiab] OR genders[tiab] OR sex[tiab] OR sexes[tiab]	**13302123**
**#5**	1 AND 2 AND 3 AND 4	**3106**
**#6**	AND ("2022/01/01"[Date - MeSH] : "3000"[Date - MeSH])	**119**
**Validation String**	27343816 OR 26873381 OR 30194636 OR 29667110 OR 31185081 OR 23141419	**6/6**

Database (including vendor/platform): Embase via Elsevier

© 2024 Westgeest AC et al. *JAMA Network Open.*

Set #	Search Strategy	Results
**#1 Staph**	'Staphylococcus aureus'/exp OR 'staphylococcus aureus':ti,ab OR 's. aureus':ti,ab OR 's aureus':ti,ab OR 'staph aureus':ti,ab	**249714**
**#2 Infection**	'bacteremia'/exp OR 'bacterial endocarditis'/exp OR bacteremia:ti,ab OR bacteraemia:ti,ab OR bacteremias:ti,ab OR bacteraemias:ti,ab OR bacteremic:ti,ab OR bacteraemic:ti,ab OR ((bloodstream:ti,ab OR 'blood stream':ti,ab OR bloodstreams:ti,ab OR 'blood streams':ti,ab) AND (infection:ti,ab OR infections:ti,ab OR infected:ti,ab OR infect:ti,ab OR infects:ti,ab OR infecting:ti,ab)) OR endocarditis:ti,ab	**153847**
**#3 Mortality**	'mortality'/de OR 'mortality rate'/exp OR mortality:ti,ab OR mortalities:ti,ab OR fatal:ti,ab OR fatality:ti,ab OR fatalities:ti,ab OR death:ti,ab OR deaths:ti,ab OR dying:ti,ab OR die:ti,ab OR died:ti,ab	**3345439**
**#4 Sex**	'female'/exp OR 'male'/exp OR 'sex difference'/exp OR female:ti,ab OR females:ti,ab OR male:ti,ab OR males:ti,ab OR women:ti,ab OR woman:ti,ab OR womens OR womans OR men:ti,ab OR gender:ti,ab OR genders:ti,ab OR sex:ti,ab OR sexes:ti,ab	**16110857**
**#5**	#1 AND #2 AND #3 AND #4	**6105**
**#6**	#1 AND #2 AND #3 AND #4 AND [humans]/lim AND ([article]/lim OR [article in press]/lim OR [conference paper]/lim)	**4138**
**#7**	#1 AND #2 AND #3 AND #4 AND [humans]/lim AND ([article]/lim OR [article in press]/lim OR [conference paper]/lim) AND [01-09-2022]/sd NOT [27-04-2023]/sd	**334**

Database (including vendor/platform): Web of Science Core Collection (1900-present) via Clarivate

Set #	Search Strategy	Results
**#1 Staph**	TS=("staphylococcus aureus" OR "s. aureus" OR "s aureus" OR "staph aureus")	**179081**
**#2 Infection**	TS=(bacteremia OR bacteraemia OR bacteremias OR bacteraemias OR bacteremic OR bacteraemic OR ((bloodstream OR "blood stream" OR bloodstreams OR "blood streams") AND (infection OR infections OR infected OR infect OR infects OR infecting)) OR endocarditis)	**102399**
**#3 Mortality**	TS=(mortality OR mortalities OR fatal OR fatality OR fatalities OR death OR deaths OR dying OR die OR died)	**2964183**

© 2024 Westgeest AC et al. *JAMA Network Open.*

**#4 Sex**	TS=(female OR females OR male OR males OR women OR woman OR womens OR womans OR men OR gender OR genders OR sex OR sexes)	**4924618**
**#5**	1 AND 2 AND 3 AND 4	**936**
**#6**	Refined by Publication Years: 2022 or 2023	**101**

eAppendix 2. Newcastle-Ottawa Quality Assessment Scale for assessing risk of bias in observational studies. Risk of bias was assessed with the Newcastle-Ottawa Assessment Scale using the questions below. The procedure for converting the responses to an overall risk of bias assessment (i.e., low, medium, or high risk of bias) is detailed here as well.

Selection

1. Representativeness of the exposed cohort

    a. Truly representative of the average patient with *S. aureus* bloodstream infection in the community (*)

    b. Somewhat representative of the average patient with *S. aureus* bloodstream infection in the community (*)

    c. Selected group of patients

    d. No description of the derivation of the cohort

2. Selection of the non-exposed cohort

    a. Drawn from the same community as the exposed cohort (*)

    b. Drawn from a different source

    c. No description of the derivation of the non-exposed cohort

3. Ascertainment of exposure

    a. Secure record (e.g. medical records) (*)

    b. Structured interview (*)

    c. Written self-report

    d. No description

4. Demonstration that outcome of interest was not present at start of study

    a. Yes (*)

    b. No

Comparability of cohorts on basis of design or analysis

1. Study controls for level of acute illness

    a. Yes (*)

    b. No

© 2024 Westgeest AC et al. *JAMA Network Open.*

2. Study controls for any additional factor.

a. Yes (*)

b. No

Outcome

1. Assessment of outcome

a. Independent blind assessment (*)

b. Record linkage (*)

c. Self-report

d. No description

2. Was follow-up long enough for outcomes to occur

a. Yes (*)

b. No

3. Adequacy of follow up of cohorts

a. Complete follow up (all subjects accounted for) (*)

b. Subjects lost to follow up unlikely to introduce bias (≤10% lost to follow-up, or description provided of those lost) (*)

c. Follow up rate < 90% and no description of those lost

d. No statement

Thresholds used to convert the Newcastle-Ottawa scale to categories (good, fair, and poor):

Good quality/low risk of bias: 3 or 4 stars in selection domain AND 1 or 2 stars in comparability domain AND 2 or 3 stars in outcome/exposure domain

Fair quality/medium risk of bias: 2 stars in selection domain AND 1 or 2 stars in comparability domain AND 2 or 3 stars in outcome/exposure domain.

Poor quality/high risk of bias: 0 or 1 star in selection domain OR 0 stars in comparability domain OR 0 or 1 stars in outcome/exposure domain

© 2024 Westgeest AC et al. *JAMA Network Open.*

eAppendix 3. Description of EPC approach.

We used the Evidence-based Practice Center (EPC) model from the U.S. Agency for Healthcare Research and Quality (AHRQ) to grade the overall strength of evidence [20]. The EPC approach evaluates the following domains: study limitations/risk of bias, consistency, directness, precision, and reporting bias. In brief, the EPC classification system applies an overall strength of evidence grade rating to an estimate effect from a body of evidence: high (we are very confident that the estimate of effect lies close to the true effect for this outcome), moderate (we are moderately confident that the estimate of effect lies close to the true effect for this outcome), low (we have limited confidence that the estimate of effect lies close to the true effect for this outcome), or insufficient (we have no evidence, we are unable to estimate an effect, or we have no confidence in the estimate of effect for this outcome). The initial strength of evidence grade was moderate given that the included observational studies in the primary adjusted analysis reduced bias from confounding through matching or statistical adjustment [20]. This baseline category could be rated down if the included studies demonstrated high risk of bias, imprecision, inconsistency, indirectness, or reporting bias.

© 2024 Westgeest AC et al. *JAMA Network Open.*

<u>eAppendix 4. Influence analysis of unadjusted mortality in patients with *Staphylococcus aureus* bacteremia.</u> An influence analysis showed that the overall results of the meta-analysis (i.e., association of female sex with increased mortality) did not change with removal of individual studies.

	OR	95%-CI	p-value	tau^2	tau	I^2
Omitting Abbas 2020	1.1193	[1.0648; 1.1766]	< 0.0001	0.0139	0.1178	37.8%
Omitting Abbas 2020	1.1201	[1.0656; 1.1774]	< 0.0001	0.0138	0.1177	37.6%
Omitting Allard 2008	1.1153	[1.0611; 1.1724]	< 0.0001	0.0139	0.1178	37.0%
Omitting Ammerlaan 2009	1.1203	[1.0657; 1.1777]	< 0.0001	0.0139	0.1179	37.6%
Omitting Asgeirsson 2011	1.1174	[1.0627; 1.1748]	< 0.0001	0.0141	0.1189	37.6%
Omitting Austin 2020	1.1119	[1.0605; 1.1659]	< 0.0001	0.0101	0.1007	31.3%
Omitting Ayau 2017	1.1242	[1.0697; 1.1814]	< 0.0001	0.0133	0.1153	36.4%
Omitting Bassetti 2017	1.1195	[1.0648; 1.1770]	< 0.0001	0.0140	0.1183	37.7%
Omitting Battle 2022	1.1238	[1.0696; 1.1807]	< 0.0001	0.0133	0.1153	36.4%
Omitting Ben-Zvi 2019	1.1172	[1.0629; 1.1744]	< 0.0001	0.0140	0.1182	37.5%
Omitting Blomfeldt 2016	1.1166	[1.0625; 1.1733]	< 0.0001	0.0138	0.1177	37.1%
Omitting Braquet 2016	1.1143	[1.0597; 1.1717]	< 0.0001	0.0139	0.1181	36.8%
Omitting Chavez 2022	1.1180	[1.0633; 1.1755]	< 0.0001	0.0142	0.1190	37.7%
Omitting Chen 2010	1.1215	[1.0667; 1.1791]	< 0.0001	0.0139	0.1180	37.3%
Omitting Chen 2015	1.1195	[1.0648; 1.1769]	< 0.0001	0.0140	0.1182	37.7%
Omitting Chen 2021	1.1191	[1.0644; 1.1766]	< 0.0001	0.0140	0.1185	37.8%
Omitting Chihara 2009	1.1179	[1.0636; 1.1750]	< 0.0001	0.0139	0.1180	37.6%
Omitting Chung 2021	1.1202	[1.0657; 1.1775]	< 0.0001	0.0138	0.1176	37.6%
Omitting Cosgrove 2005	1.1229	[1.0688; 1.1797]	< 0.0001	0.0134	0.1159	36.6%
Omitting Eells 2013	1.1144	[1.0622; 1.1691]	< 0.0001	0.0130	0.1140	34.5%
Omitting Forsblom 2018	1.1203	[1.0656; 1.1778]	< 0.0001	0.0139	0.1181	37.6%
Omitting Gasch 2013	1.1181	[1.0633; 1.1757]	< 0.0001	0.0142	0.1192	37.7%
Omitting Greenberg 2014	1.1191	[1.0645; 1.1765]	< 0.0001	0.0140	0.1182	37.8%
Omitting Guillamet 2018	1.1180	[1.0636; 1.1753]	< 0.0001	0.0140	0.1181	37.6%
Omitting Hagstrand Aldman 2022	1.1190	[1.0643; 1.1764]	< 0.0001	0.0140	0.1184	37.8%
Omitting HallIi 2012	1.1227	[1.0705; 1.1775]	< 0.0001	0.0133	0.1155	34.1%
Omitting Han 2012	1.1163	[1.0625; 1.1729]	< 0.0001	0.0138	0.1174	36.8%
Omitting Honda 2010	1.1181	[1.0635; 1.1754]	< 0.0001	0.0140	0.1183	37.7%
Omitting Horvath 2020	1.1176	[1.0630; 1.1749]	< 0.0001	0.0141	0.1185	37.6%
Omitting Hsu 2007	1.1234	[1.0698; 1.1797]	< 0.0001	0.0132	0.1151	35.9%
Omitting Jokinen 2017	1.1185	[1.0638; 1.1760]	< 0.0001	0.0141	0.1186	37.8%
Omitting Joo 2013	1.1227	[1.0687; 1.1795]	< 0.0001	0.0134	0.1160	36.6%
Omitting Joost 2017	1.1214	[1.0662; 1.1795]	< 0.0001	0.0142	0.1191	37.2%
Omitting Jorgensen 2019	1.1172	[1.0628; 1.1744]	< 0.0001	0.0140	0.1183	37.5%
Omitting Kang 2018	1.1207	[1.0655; 1.1787]	< 0.0001	0.0143	0.1196	37.4%
Omitting Kempker 2010	1.1216	[1.0668; 1.1793]	< 0.0001	0.0139	0.1180	37.3%
Omitting Kim 2008	1.1200	[1.0654; 1.1774]	< 0.0001	0.0139	0.1180	37.6%
Omitting Kim 2010	1.1196	[1.0648; 1.1772]	< 0.0001	0.0141	0.1188	37.7%
Omitting Kim 2019	1.1224	[1.0682; 1.1793]	< 0.0001	0.0135	0.1163	36.8%
Omitting Kim 2019	1.1205	[1.0659; 1.1779]	< 0.0001	0.0139	0.1178	37.5%
Omitting Kobayashi 2014	1.1187	[1.0640; 1.1762]	< 0.0001	0.0141	0.1187	37.8%
Omitting Lamagni 2011	1.1204	[1.0646; 1.1791]	< 0.0001	0.0148	0.1215	36.4%
Omitting Laupland 2022	1.1179	[1.0621; 1.1766]	< 0.0001	0.0151	0.1228	37.6%
Omitting Lee 2013	1.1184	[1.0638; 1.1757]	< 0.0001	0.0140	0.1183	37.7%
Omitting Lee 2021	1.1216	[1.0675; 1.1784]	< 0.0001	0.0136	0.1167	36.9%
Omitting Lee 2021	1.1196	[1.0648; 1.1772]	< 0.0001	0.0141	0.1187	37.7%
Omitting Lesens 2006	1.1200	[1.0655; 1.1772]	< 0.0001	0.0138	0.1176	37.6%
Omitting Mansur 2012	1.1130	[1.0596; 1.1691]	< 0.0001	0.0127	0.1127	35.3%
Omitting Mejer 2012	1.1185	[1.0628; 1.1772]	< 0.0001	0.0150	0.1225	37.5%
Omitting Mejer 2012	1.1128	[1.0585; 1.1699]	< 0.0001	0.0133	0.1152	34.9%
Omitting Melzer 2013	1.1239	[1.0700; 1.1805]	< 0.0001	0.0132	0.1149	36.1%
Omitting Molkanen 2016	1.1200	[1.0654; 1.1774]	< 0.0001	0.0139	0.1180	37.6%
Omitting Murdoch 2017	1.1158	[1.0609; 1.1737]	< 0.0001	0.0144	0.1201	37.5%
Omitting Nambiar 2018	1.1212	[1.0659; 1.1793]	< 0.0001	0.0143	0.1196	37.2%
Omitting Osthoff 2016	1.1292	[1.0770; 1.1838]	< 0.0001	0.0116	0.1075	32.8%
Omitting Papadimitriou-Olivgeris 2023	1.1208	[1.0662; 1.1781]	< 0.0001	0.0138	0.1176	37.5%
Omitting Park 2015	1.1224	[1.0680; 1.1794]	< 0.0001	0.0136	0.1165	36.9%
Omitting Paulsen 2015	1.1182	[1.0636; 1.1757]	< 0.0001	0.0141	0.1187	37.7%
Omitting Perovic 2006	1.1213	[1.0668; 1.1787]	< 0.0001	0.0138	0.1175	37.3%
Omitting Rieg 2009	1.1196	[1.0648; 1.1772]	< 0.0001	0.0141	0.1186	37.7%
Omitting Roth 2017	1.1182	[1.0635; 1.1757]	< 0.0001	0.0141	0.1188	37.7%
Omitting Saunderson 2015	1.1190	[1.0643; 1.1765]	< 0.0001	0.0141	0.1186	37.8%
Omitting Seas 2018	1.1178	[1.0629; 1.1754]	< 0.0001	0.0143	0.1195	37.7%
Omitting Smit 2017	1.1124	[1.0591; 1.1682]	< 0.0001	0.0121	0.1101	34.2%
Omitting Soriano 2000	1.1168	[1.0623; 1.1741]	< 0.0001	0.0141	0.1186	37.5%
Omitting Soriano 2008	1.1179	[1.0633; 1.1753]	< 0.0001	0.0141	0.1186	37.7%
Omitting Sullivan 2017	1.1165	[1.0627; 1.1730]	< 0.0001	0.0138	0.1174	36.8%
Omitting Szubert 2019	1.1209	[1.0665; 1.1782]	< 0.0001	0.0138	0.1174	37.4%
Omitting Tan 2021	1.1170	[1.0628; 1.1740]	< 0.0001	0.0139	0.1179	37.3%
Omitting Ternavasio-de la Vega 2018	1.1219	[1.0676; 1.1788]	< 0.0001	0.0136	0.1167	37.0%
Omitting Thorlacius-Ussing 2019	1.1124	[1.0580; 1.1696]	< 0.0001	0.0131	0.1146	32.4%
Omitting Thwaites 2010	1.1187	[1.0639; 1.1763]	< 0.0001	0.0142	0.1190	37.8%
Omitting Tong 2012	1.1234	[1.0681; 1.1816]	< 0.0001	0.0139	0.1178	36.0%
Omitting Turnidge 2007	1.1185	[1.0638; 1.1761]	< 0.0001	0.0141	0.1187	37.8%
Omitting Turnidge 2009	1.1242	[1.0695; 1.1818]	< 0.0001	0.0134	0.1158	36.4%
Omitting vanHal 2011	1.1182	[1.0635; 1.1757]	< 0.0001	0.0141	0.1187	37.7%
Omitting Wang 2008	1.1168	[1.0634; 1.1730]	< 0.0001	0.0138	0.1173	36.5%
Omitting Wang 2013	1.1187	[1.0641; 1.1761]	< 0.0001	0.0140	0.1182	37.8%
Omitting Wang 2015	1.1224	[1.0680; 1.1795]	< 0.0001	0.0136	0.1166	37.0%
Omitting Wi 2018	1.1214	[1.0669; 1.1787]	< 0.0001	0.0138	0.1173	37.3%
Omitting Willekens 2021	1.1176	[1.0630; 1.1749]	< 0.0001	0.0140	0.1185	37.6%
Omitting Yilmaz 2016	1.1209	[1.0666; 1.1780]	< 0.0001	0.0137	0.1172	37.3%
Omitting Yoon 2016	1.1246	[1.0714; 1.1804]	< 0.0001	0.0130	0.1138	35.2%
Pooled estimate	1.1193	[1.0652; 1.1761]	< 0.0001	0.0138	0.1174	37.0%

© 2024 Westgeest AC et al. *JAMA Network Open.*

eAppendix 5. Influence analysis of adjusted mortality in patients with *Staphylococcus aureus* bacteremia. An influence analysis showed that the overall results of the meta-analysis (i.e., association of female sex with increased mortality) did not change with removal of individual studies.

	OR	95%-CI	p-value	tau^2	tau	I^2
Omitting Allard 2008	1.1757	[1.0998; 1.2568]	< 0.0001	0.0117	0.1084	50.0%
Omitting Austin 2020	1.1619	[1.0920; 1.2363]	< 0.0001	0.0081	0.0898	44.1%
Omitting Bai 2015	1.1784	[1.1021; 1.2600]	< 0.0001	0.0121	0.1102	50.7%
Omitting Benfield 2007	1.1956	[1.1181; 1.2785]	< 0.0001	0.0109	0.1045	48.1%
Omitting Benfield 2007	1.1956	[1.1164; 1.2805]	< 0.0001	0.0117	0.1084	47.4%
Omitting Blomfeldt 2016	1.1840	[1.1053; 1.2683]	< 0.0001	0.0128	0.1132	52.2%
Omitting Braquet 2016	1.1780	[1.0993; 1.2623]	< 0.0001	0.0127	0.1127	50.8%
Omitting Chen 2010	1.1911	[1.1132; 1.2744]	< 0.0001	0.0125	0.1119	51.1%
Omitting Cobussen 2018	1.1840	[1.1057; 1.2678]	< 0.0001	0.0126	0.1124	52.2%
Omitting Eells 2013	1.1753	[1.1026; 1.2528]	< 0.0001	0.0114	0.1069	48.3%
Omitting Forsblom 2018	1.1838	[1.1047; 1.2685]	< 0.0001	0.0130	0.1140	52.2%
Omitting Gasch 2013	1.1796	[1.1016; 1.2632]	< 0.0001	0.0126	0.1123	51.4%
Omitting Joo 2013	1.1837	[1.1051; 1.2678]	< 0.0001	0.0128	0.1130	52.2%
Omitting Kang 2018	1.1923	[1.1158; 1.2740]	< 0.0001	0.0120	0.1094	50.1%
Omitting Lamagni 2011	1.1953	[1.1155; 1.2807]	< 0.0001	0.0122	0.1103	48.1%
Omitting Laupland 2022	1.1884	[1.1061; 1.2769]	< 0.0001	0.0146	0.1209	52.2%
Omitting Lee 2021	1.1845	[1.1054; 1.2693]	< 0.0001	0.0130	0.1142	52.2%
Omitting Mansur 2012	1.1715	[1.0967; 1.2514]	< 0.0001	0.0108	0.1041	48.6%
Omitting Maor 2009	1.1775	[1.1024; 1.2577]	< 0.0001	0.0119	0.1092	50.0%
Omitting Mejer 2012	1.1911	[1.1089; 1.2793]	< 0.0001	0.0143	0.1196	51.9%
Omitting Mejer 2012	1.1833	[1.1016; 1.2711]	< 0.0001	0.0143	0.1196	51.0%
Omitting Meredith 2021	1.1864	[1.1076; 1.2708]	< 0.0001	0.0129	0.1138	52.1%
Omitting Murdoch 2017	1.1853	[1.1047; 1.2718]	< 0.0001	0.0138	0.1174	52.2%
Omitting Nambiar 2018	1.1850	[1.1042; 1.2718]	< 0.0001	0.0139	0.1180	52.1%
Omitting Osthoff 2016	1.1890	[1.1110; 1.2725]	< 0.0001	0.0128	0.1129	51.5%
Omitting Rieg 2013	1.1873	[1.1102; 1.2697]	< 0.0001	0.0126	0.1124	51.2%
Omitting Saunderson 2015	1.1867	[1.1080; 1.2710]	< 0.0001	0.0129	0.1137	52.0%
Omitting Schneider 2020	1.1820	[1.1034; 1.2661]	< 0.0001	0.0128	0.1132	51.9%
Omitting Smit 2017	1.1757	[1.0974; 1.2596]	< 0.0001	0.0123	0.1110	49.8%
Omitting Soriano 2000	1.1776	[1.1015; 1.2588]	< 0.0001	0.0120	0.1096	50.4%
Omitting Szubert 2019	1.1887	[1.1127; 1.2698]	< 0.0001	0.0125	0.1117	50.4%
Omitting Thorlacius-Ussing 2019	1.1848	[1.1028; 1.2729]	< 0.0001	0.0145	0.1204	51.5%
Omitting Thwaites 2010	1.1876	[1.1087; 1.2722]	< 0.0001	0.0130	0.1142	52.0%
Omitting Yahav 2017	1.1762	[1.0989; 1.2589]	< 0.0001	0.0121	0.1100	50.4%
Pooled estimate	1.1836	[1.1067; 1.2658]	< 0.0001	0.0125	0.1119	50.7%

© 2024 Westgeest AC et al. *JAMA Network Open.*

eTable 1. Newcastle-Ottawa quality assessment of individual studies

The Newcastle-Ottawa Quality Assessment Scale determines a study's risk of bias through nine questions (detailed in Appendix 2). For each study, the grades for the nine questions are shown below. Grades that receive a star are highlighted in green, while those that do not are highlighted in red. Based on the grades from each question in the Newcastle-Ottawa Scale, an overall risk of bias (high, medium, low) can be assigned (detailed in Appendix 2).

Study	**Selection:** Representativeness of the exposed cohort	**Selection:** Selection of the non-exposed cohort	**Selection:** Ascertainment of exposure	**Selection:** Outcome of interest not present at start	**Comparability:** Study controls for level of acute illness	**Comparability:** Study controls for any additional factor	**Outcome:** Assessment of outcome	**Outcome:** Follow-up long enough for outcomes to occur	**Outcome:** Adequacy of follow up of cohorts	**Risk of bias**
Abbas 2020	b	a	a	a	b	b	b	a	a	Poor
Allard 2008	b	a	a	a	b	a	b	a	a	Good
Ammerlaan 2009	a	a	a	a	b	b	b	a	a	Poor
Asgeirsson 2011	b	a	a	a	b	b	b	a	a	Poor
Austin 2020	b	a	a	a	a	a	b	a	a	Good
Ayau 2017	c	a	a	a	b	b	b	a	a	Poor
Bai 2015	c	a	a	a	a	a	b	a	a	Good
Bassetti 2017	b	a	a	a	b	b	b	a	a	Poor
Battle 2022	b	a	a	a	b	b	b	a	a	Poor
Ben-Zvi 2019	b	a	a	a	b	b	b	a	a	Poor
Benfield 2007	b	a	a	a	b	a	b	a	b	Good
Blomfeldt 2016	b	a	a	a	b	a	b	a	d	Good
Braquet 2016	b	a	a	a	a	a	b	a	b	Good
Chavez 2022	b	a	a	a	b	b	b	a	d	Poor
Chen 2010	b	a	a	a	a	a	b	a	a	Good
Chen 2015	b	a	a	a	b	b	b	a	b	Poor
Chen 2021	c	a	a	a	b	b	b	a	a	Poor
Chihara 2009	c	a	a	a	b	b	b	a	a	Poor
Chung 2021	c	a	a	a	b	b	b	a	a	Poor
Cobussen 2018	b	a	a	a	a	a	b	a	a	Good
Cosgrove 2005	b	a	a	a	b	b	b	a	a	Poor
Eells 2013	c	a	a	a	a	a	b	a	a	Good

© 2024 Westgeest AC et al. *JAMA Network Open.*

Forsblom 2018	c	a	a	a	a	a	b	a	b	Good
Gasch 2013	c	a	a	a	a	a	b	a	b	Good
Greenberg 2014	b	a	a	a	b	b	b	a	a	Poor
Guillamet 2018	c	a	a	a	b	b	b	a	b	Poor
HagstrandAldman 2022	c	a	a	a	b	b	b	a	a	Poor
Hallli 2012	c	a	a	a	b	b	b	a	a	Poor
Han 2012	b	a	a	a	b	b	b	a	a	Poor
Honda 2010	b	a	a	a	b	b	b	a	a	Poor
Horváth 2020	b	a	a	a	b	b	b	a	a	Poor
Hsu 2007	c	a	a	a	b	b	b	a	a	Poor
Jokinen 2017	b	a	a	a	b	b	b	a	c	Poor
Joo 2013	c	a	a	a	a	a	b	a	a	Good
Joost 2017	b	a	a	a	b	b	b	a	d	Poor
Jorgensen 2019	c	a	a	a	b	b	b	a	a	Poor
Kang 2018	b	a	a	a	a	a	b	a	d	Good
Kempker 2010	c	a	a	a	b	b	b	a	a	Poor
Kim 2008	b	a	a	a	b	b	b	a	a	Poor
Kim 2010	b	a	a	a	b	b	b	a	a	Poor
Kim 2019	b	a	a	a	b	b	b	a	d	Poor
Kim 2019	b	a	a	a	b	b	b	a	a	Poor
Kobayashi 2014	b	a	a	a	b	b	b	a	a	Poor
Lamagni 2011	c	a	a	a	a	a	b	a	a	Good
Laupland 2022	b	a	a	a	a	a	b	a	d	Good
Lee 2013	c	a	a	a	b	b	b	a	a	Poor
Lee 2021	c	a	a	a	b	a	b	a	a	Good
Lee 2021	c	a	a	a	b	b	b	a	a	Poor
Lesens 2006	a	a	a	a	b	b	b	a	a	Poor
Mansur 2012	b	a	a	a	a	a	b	a	a	Good
Maor 2009	c	a	a	a	b	a	b	a	d	Good
Mejer 2012	b	a	a	a	b	a	b	a	b	Good
Melzer 2013	c	a	a	a	b	b	b	a	a	Poor
Meredith 2021	b	a	a	a	a	a	b	a	d	Good
Mölkänen 2016	b	a	a	a	b	b	b	a	d	Poor
Murdoch 2017	b	a	a	a	b	a	b	a	d	Good
Nambiar 2018	a	a	a	a	a	a	b	a	d	Good
Osthoff 2016	b	a	a	a	b	a	b	a	d	Good
Papadimitriou-Olivgeris	b	a	a	a	b	b	b	a	d	Poor
Park 2015	b	a	a	a	b	b	b	a	d	Poor
Paulsen 2015	b	a	a	a	b	b	b	a	a	Poor
Perovic 2006	b	a	a	a	b	b	b	a	b	Poor
Rieg 2009	b	a	a	a	b	b	b	a	a	Poor
Rieg 2013	b	a	a	a	b	a	b	a	d	Good
Roth 2017	b	a	a	a	b	b	b	a	b	Poor
Saunderson 2015	b	a	a	a	b	a	b	a	a	Good
Schneider 2020	b	a	a	a	b	a	b	a	a	Good
Seas 2018	a	a	a	a	b	b	b	a	b	Poor
Smit 2017	b	a	a	a	a	a	b	a	a	Good

© 2024 Westgeest AC et al. *JAMA Network Open.*

Soriano 2000	b	a	a	a	a	a	b	a	a	Good
Soriano 2008	c	a	a	a	b	b	b	a	a	Poor
Sullivan 2017	c	a	a	a	b	b	b	a	d	Poor
Szubert 2019	b	a	a	a	a	a	b	a	d	Good
Tan 2021	b	a	a	a	b	b	b	a	d	Poor
Ternavasio-delaVega	b	a	a	a	b	b	b	a	a	Poor
Thorlacius-Ussing 2019	b	a	a	a	b	a	b	a	a	Good
Thwaites 2010	a	a	a	a	b	a	b	a	b	Good
Tong 2012	b	a	a	a	b	b	b	a	a	Poor
Turnidge 2007	b	a	a	a	b	b	b	a	c	Poor
Turnidge 2009	b	a	a	a	b	b	b	a	a	Poor
vanHal 2011	b	a	a	a	b	b	b	a	a	Poor
Wang 2008	c	a	a	a	b	b	b	a	a	Poor
Wang 2013	b	c	a	a	b	b	b	a	a	Poor
Wang 2015	c	a	a	a	b	b	b	a	a	Poor
Wi 2018	a	c	a	a	b	b	b	a	d	Poor
Willekens 2021	b	a	a	a	b	b	b	a	a	Poor
Yahav 2017	b	c	a	a	b	a	b	a	a	Good
Yilmaz 2016	b	a	a	a	b	b	b	a	d	Poor
Yoon 2016	b	a	a	a	b	b	b	a	d	Poor

© 2024 Westgeest AC et al. *JAMA Network Open.*

eFigure 1. Sensitivity analysis of unadjusted mortality. Only studies that either directly reported an odds ratio (OR) or contained raw mortality data such that ORs could be directly calculated are included here. Studies that reported a hazard ratio, relative risk, or mortality rate ratio were excluded.

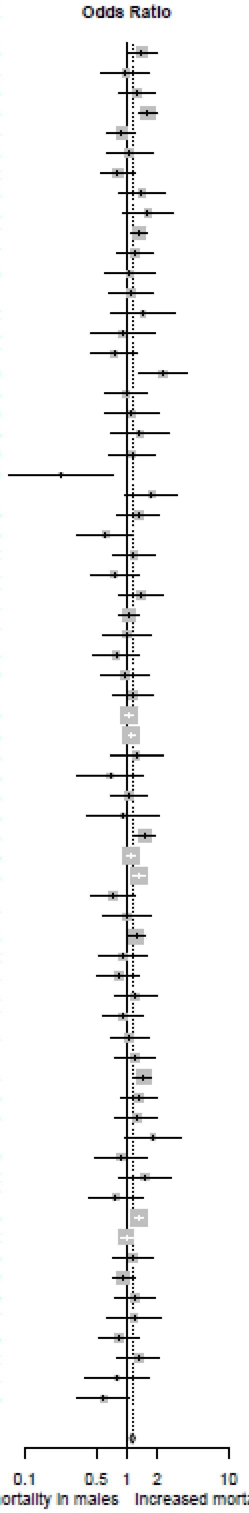

Study	TE	seTE	OR	95%-CI	Weight
Allard 2008	0.34	0.1665	1.40	[1.01; 1.94]	1.9%
Ammerlaan 2009	−0.03	0.2734	0.97	[0.57; 1.67]	0.9%
Asgeirsson 2011	0.23	0.2035	1.26	[0.85; 1.88]	1.4%
Austin 2020	0.47	0.0968	1.60	[1.32; 1.93]	3.3%
Ayau 2017	−0.12	0.1564	0.88	[0.65; 1.20]	2.0%
Bassetti 2017	0.06	0.2608	1.06	[0.64; 1.77]	0.9%
Battle 2022	−0.20	0.2032	0.82	[0.55; 1.22]	1.4%
Ben−Zvi 2019	0.34	0.2718	1.41	[0.83; 2.40]	0.9%
Blomfeldt 2016	0.47	0.2858	1.60	[0.91; 2.80]	0.8%
Braquet 2016	0.27	0.0987	1.31	[1.08; 1.59]	3.2%
Chavez 2022	0.19	0.2097	1.20	[0.80; 1.82]	1.3%
Chen 2015	0.06	0.2830	1.06	[0.61; 1.85]	0.8%
Chen 2021	0.11	0.2511	1.11	[0.68; 1.82]	1.0%
Chihara 2009	0.37	0.3682	1.45	[0.71; 2.99]	0.5%
Chung 2021	−0.10	0.3718	0.91	[0.44; 1.88]	0.5%
Cosgrove 2005	−0.28	0.2667	0.76	[0.45; 1.28]	0.9%
Eells 2013	0.82	0.2699	2.28	[1.34; 3.87]	0.9%
Forsblom 2018	−0.01	0.2476	0.99	[0.61; 1.61]	1.0%
Greenberg 2014	0.12	0.3160	1.12	[0.60; 2.09]	0.7%
Guillamet 2018	0.30	0.3283	1.35	[0.71; 2.56]	0.6%
Hagstrand Aldman 2022	0.12	0.2659	1.13	[0.67; 1.90]	0.9%
Halili 2012	−1.49	0.6050	0.23	[0.07; 0.74]	0.2%
Han 2012	0.55	0.3031	1.74	[0.96; 3.15]	0.7%
Horvath 2020	0.26	0.2443	1.30	[0.80; 2.10]	1.1%
Hsu 2007	−0.48	0.3185	0.62	[0.33; 1.16]	0.7%
Jokinen 2017	0.16	0.2462	1.18	[0.73; 1.91]	1.0%
Joo 2013	−0.29	0.2764	0.75	[0.44; 1.29]	0.9%
Jorgensen 2019	0.32	0.2567	1.37	[0.83; 2.27]	1.0%
Kang 2018	0.04	0.1127	1.04	[0.84; 1.30]	2.9%
Kim 2008	0.00	0.2787	1.00	[0.58; 1.73]	0.8%
Kim 2019	−0.24	0.2708	0.79	[0.46; 1.34]	0.9%
Kim 2019	−0.05	0.2700	0.95	[0.56; 1.61]	0.9%
Kobayashi 2014	0.14	0.2341	1.15	[0.73; 1.82]	1.1%
Lamagni 2011	0.07	0.0446	1.07	[0.98; 1.16]	4.7%
Laupland 2022	0.11	0.0425	1.12	[1.03; 1.22]	4.8%
Lee 2013	0.21	0.2985	1.24	[0.69; 2.22]	0.8%
Lee 2021	−0.37	0.3795	0.69	[0.33; 1.46]	0.5%
Lee 2021	0.06	0.2125	1.06	[0.70; 1.61]	1.3%
Lesens 2006	−0.08	0.4106	0.92	[0.41; 2.07]	0.4%
Mansur 2012	0.39	0.1155	1.48	[1.18; 1.86]	2.8%
Mejer 2012	0.10	0.0483	1.11	[1.01; 1.22]	4.6%
Mejer 2012	0.28	0.0635	1.33	[1.17; 1.50]	4.2%
Melzer 2013	−0.31	0.2481	0.73	[0.45; 1.19]	1.0%
Moikanen 2016	0.00	0.2787	1.00	[0.58; 1.73]	0.8%
Murdoch 2017	0.21	0.1068	1.23	[1.00; 1.52]	3.0%
Papadimitriou−Olivgeris 2023	−0.09	0.2812	0.92	[0.53; 1.59]	0.8%
Park 2015	−0.18	0.2434	0.83	[0.52; 1.34]	1.1%
Paulsen 2015	0.19	0.2424	1.21	[0.75; 1.95]	1.1%
Perovic 2006	−0.09	0.2337	0.92	[0.58; 1.45]	1.1%
Rieg 2009	0.06	0.2204	1.06	[0.69; 1.63]	1.2%
Roth 2017	0.19	0.2291	1.20	[0.77; 1.89]	1.2%
Smit 2017	0.38	0.0914	1.46	[1.22; 1.74]	3.4%
Soriano 2000	0.29	0.2086	1.33	[0.89; 2.01]	1.3%
Soriano 2008	0.22	0.2435	1.25	[0.78; 2.01]	1.1%
Sullivan 2017	0.60	0.3311	1.81	[0.95; 3.47]	0.6%
Szubert 2019	−0.12	0.2969	0.88	[0.49; 1.58]	0.8%
Tan 2021	0.42	0.2972	1.52	[0.85; 2.72]	0.8%
Ternavasio−de la Vega 2018	−0.26	0.3077	0.77	[0.42; 1.42]	0.7%
Thorlacius−Ussing 2019	0.27	0.0455	1.32	[1.20; 1.44]	4.7%
Tong 2012	0.00	0.0742	1.00	[0.86; 1.16]	3.9%
Turnidge 2007	0.15	0.2345	1.17	[0.74; 1.85]	1.1%
Turnidge 2009	−0.06	0.1199	0.94	[0.74; 1.19]	2.7%
vanHal 2011	0.19	0.2343	1.21	[0.76; 1.91]	1.1%
Wang 2013	0.17	0.3117	1.19	[0.65; 2.19]	0.7%
Wang 2015	−0.16	0.2333	0.85	[0.54; 1.34]	1.1%
Willekens 2021	0.26	0.2462	1.30	[0.80; 2.11]	1.0%
Yilmaz 2016	−0.22	0.3674	0.80	[0.39; 1.65]	0.5%
Yoon 2016	−0.53	0.2953	0.59	[0.33; 1.05]	0.8%
**Random effects model**			**1.14**	**[1.08; 1.20]**	**100.0%**

Heterogeneity: $I^2 = 40\%$, $\tau^2 = 0.0147$, $p < 0.01$

© 2024 Westgeest AC et al. *JAMA Network Open.*

<u>eFigure 2. Funnel plot of studies included in the analysis of unadjusted mortality.</u> One study in particular had an effect size that was larger than expected based on the standard error (lower left corner of plot). This study demonstrated significantly lower mortality in females relative to males. Despite this, Egger's test did not reveal significant asymmetry in the funnel plot (p=0.06). Thus in total no clear evidence of publication bias was detected.

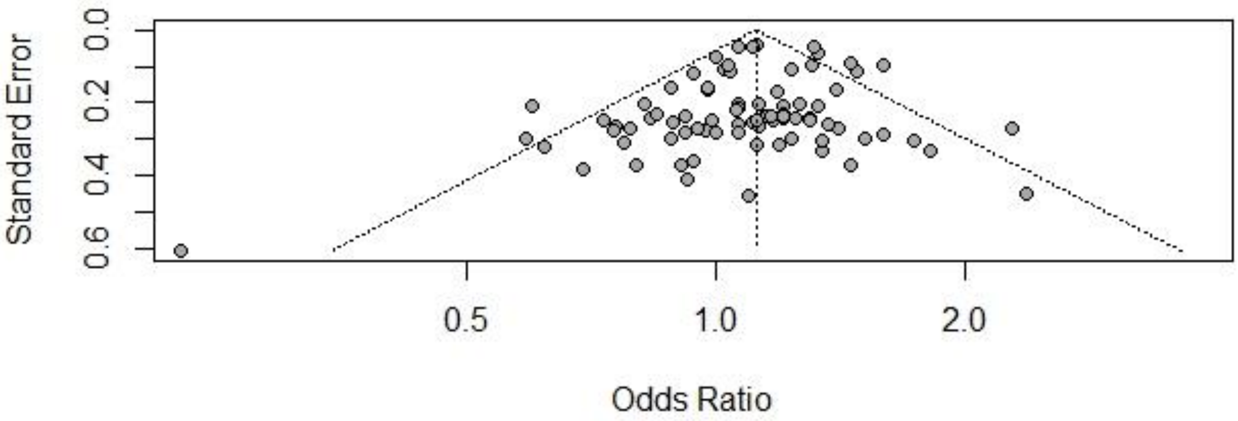

© 2024 Westgeest AC et al. *JAMA Network Open.*

eAppendix 5. Influence analysis of adjusted mortality in patients with *Staphylococcus aureus* bacteremia. An influence analysis showed that the overall results of the meta-analysis (i.e., association of female sex with increased mortality) did not change with removal of individual studies.

	OR	95%-CI	p-value	tau^2	tau	I^2
Omitting Allard 2008	1.1757	[1.0998; 1.2568]	< 0.0001	0.0117	0.1084	50.0%
Omitting Austin 2020	1.1619	[1.0920; 1.2363]	< 0.0001	0.0081	0.0898	44.1%
Omitting Bai 2015	1.1784	[1.1021; 1.2600]	< 0.0001	0.0121	0.1102	50.7%
Omitting Benfield 2007	1.1956	[1.1181; 1.2785]	< 0.0001	0.0109	0.1045	48.1%
Omitting Benfield 2007	1.1956	[1.1164; 1.2805]	< 0.0001	0.0117	0.1084	47.4%
Omitting Blomfeldt 2016	1.1840	[1.1053; 1.2683]	< 0.0001	0.0128	0.1132	52.2%
Omitting Braquet 2016	1.1780	[1.0993; 1.2623]	< 0.0001	0.0127	0.1127	50.8%
Omitting Chen 2010	1.1911	[1.1132; 1.2744]	< 0.0001	0.0125	0.1119	51.1%
Omitting Cobussen 2018	1.1840	[1.1057; 1.2678]	< 0.0001	0.0126	0.1124	52.2%
Omitting Eells 2013	1.1753	[1.1026; 1.2528]	< 0.0001	0.0114	0.1069	48.3%
Omitting Forsblom 2018	1.1838	[1.1047; 1.2685]	< 0.0001	0.0130	0.1140	52.2%
Omitting Gasch 2013	1.1796	[1.1016; 1.2632]	< 0.0001	0.0126	0.1123	51.4%
Omitting Joo 2013	1.1837	[1.1051; 1.2678]	< 0.0001	0.0128	0.1130	52.2%
Omitting Kang 2018	1.1923	[1.1158; 1.2740]	< 0.0001	0.0120	0.1094	50.1%
Omitting Lamagni 2011	1.1953	[1.1155; 1.2807]	< 0.0001	0.0122	0.1103	48.1%
Omitting Laupland 2022	1.1884	[1.1061; 1.2769]	< 0.0001	0.0146	0.1209	52.2%
Omitting Lee 2021	1.1845	[1.1054; 1.2693]	< 0.0001	0.0130	0.1142	52.2%
Omitting Mansur 2012	1.1715	[1.0967; 1.2514]	< 0.0001	0.0108	0.1041	48.6%
Omitting Maor 2009	1.1775	[1.1024; 1.2577]	< 0.0001	0.0119	0.1092	50.0%
Omitting Mejer 2012	1.1911	[1.1089; 1.2793]	< 0.0001	0.0143	0.1196	51.9%
Omitting Mejer 2012	1.1833	[1.1016; 1.2711]	< 0.0001	0.0143	0.1196	51.0%
Omitting Meredith 2021	1.1864	[1.1076; 1.2708]	< 0.0001	0.0129	0.1138	52.1%
Omitting Murdoch 2017	1.1853	[1.1047; 1.2718]	< 0.0001	0.0138	0.1174	52.2%
Omitting Nambiar 2018	1.1850	[1.1042; 1.2718]	< 0.0001	0.0139	0.1180	52.1%
Omitting Osthoff 2016	1.1890	[1.1110; 1.2725]	< 0.0001	0.0128	0.1129	51.5%
Omitting Rieg 2013	1.1873	[1.1102; 1.2697]	< 0.0001	0.0126	0.1124	51.2%
Omitting Saunderson 2015	1.1867	[1.1080; 1.2710]	< 0.0001	0.0129	0.1137	52.0%
Omitting Schneider 2020	1.1820	[1.1034; 1.2661]	< 0.0001	0.0128	0.1132	51.9%
Omitting Smit 2017	1.1757	[1.0974; 1.2596]	< 0.0001	0.0123	0.1110	49.8%
Omitting Soriano 2000	1.1776	[1.1015; 1.2588]	< 0.0001	0.0120	0.1096	50.4%
Omitting Szubert 2019	1.1887	[1.1127; 1.2698]	< 0.0001	0.0125	0.1117	50.4%
Omitting Thorlacius-Ussing 2019	1.1848	[1.1028; 1.2729]	< 0.0001	0.0145	0.1204	51.5%
Omitting Thwaites 2010	1.1876	[1.1087; 1.2722]	< 0.0001	0.0130	0.1142	52.0%
Omitting Yahav 2017	1.1762	[1.0989; 1.2589]	< 0.0001	0.0121	0.1100	50.4%
Pooled estimate	1.1836	[1.1067; 1.2658]	< 0.0001	0.0125	0.1119	50.7%

© 2024 Westgeest AC et al. *JAMA Network Open.*

eFigure 3. Sensitivity analysis adjusted mortality. Only studies that either directly reported an odds ratio (OR) or contained raw mortality data such that ORs could be directly calculated are included here. Studies that reported a hazard ratio, relative risk, or mortality rate ratio were excluded.

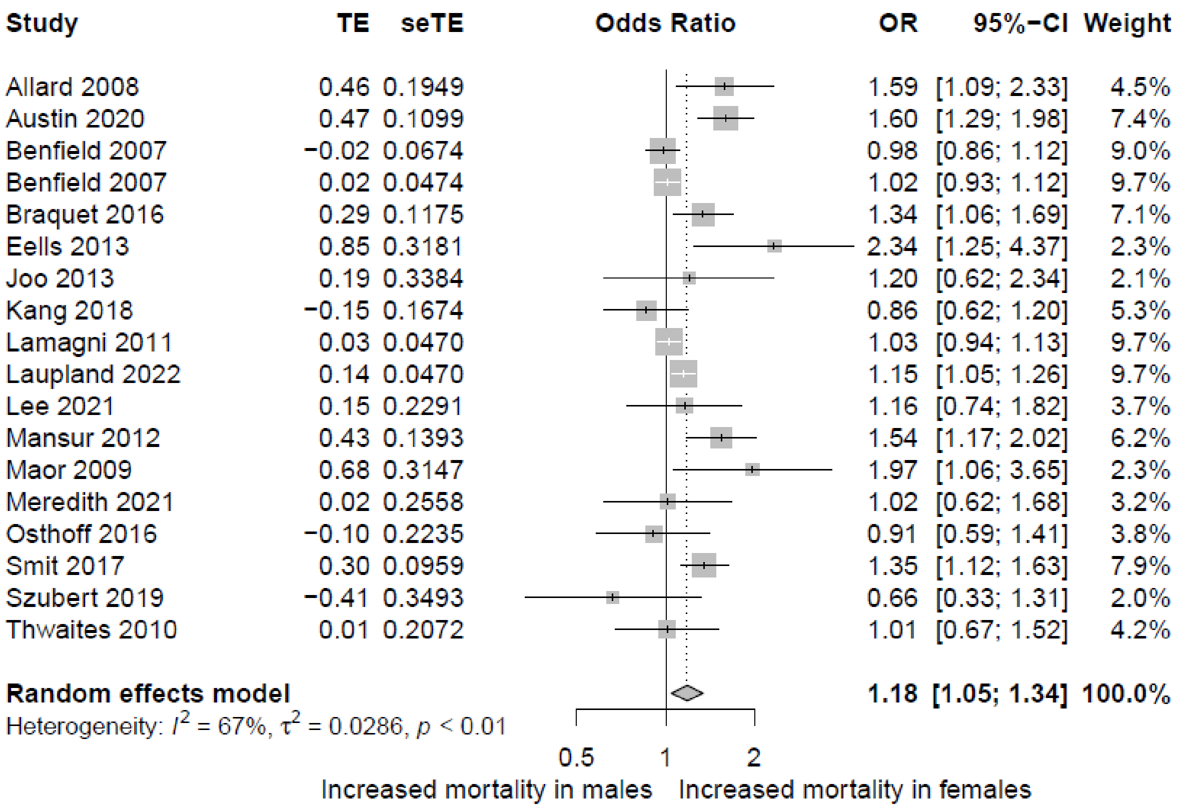

© 2024 Westgeest AC et al. *JAMA Network Open.*

eFigure 4. Funnel plot for studies included in analysis of adjusted mortality. Egger's test did not reveal significant asymmetry in the funnel plot (p=0.10).

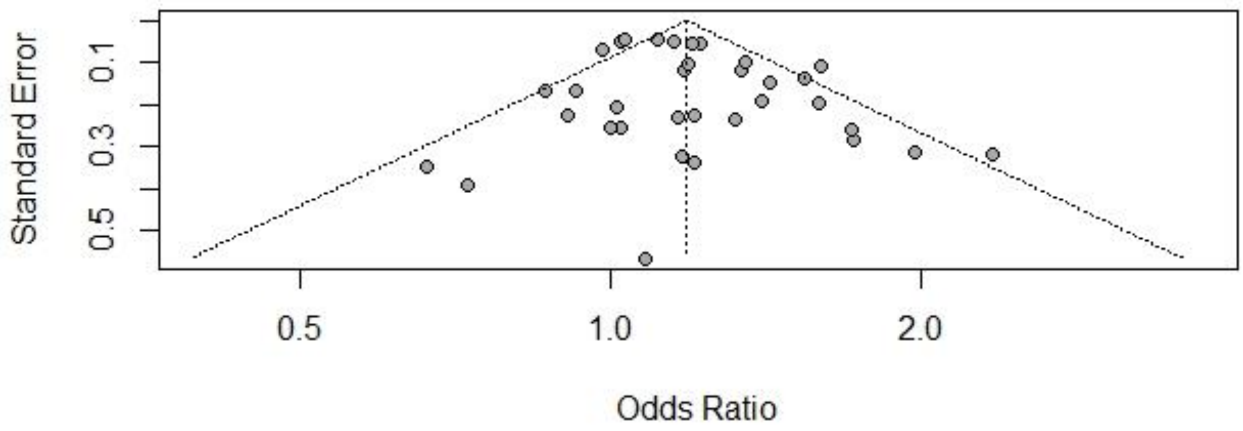

© 2024 Westgeest AC et al. *JAMA Network Open.*

eTable 2. Evidence profile for association of female sex and mortality in patients with *Staphylococcus aureus* bacteremia.

**Patient population**	Patients with *Staphylococcus aureus* bacteremia
**Setting**	Hospital
**Intervention**	Female sex
**Comparison**	Male sex
**Outcome**	Mortality
**Studies (participants)**	89 (132,582)
**Risk of bias**	High
**Consistency**	Consistent
**Precision**	Precise
**Directness**	Direct
**Other limitations**	Sex-difference was not the primary outcome of interest in the majority of the studies that were included
**Overall strength of evidence**	Low
**Conclusion**	Female sex is associated with higher mortality in patients with SAB
**Summary estimate**	Unadjusted: 1.12 (95%CI 1.07-1.18)<br>Adjusted: 1.18 (95%CI 1.11-1.27)

© 2024 Westgeest AC et al. *JAMA Network Open.*
