# Supplementary material for: Female Sex and Mortality in Patients with Staphylococcus aureus Bacteremia: A Systematic Review and Meta-analysis
Source: JAMA Netw Open. 2024 Feb 27;7(2):e240473. doi: 10.1001/jamanetworkopen.2024.0473 (PMC10900971; doi:10.1001/jamanetworkopen.2024.0473)
Supplement: Supplement 2. — Data Sharing Statement [file jamanetwopen-e240473-s002.pdf]

Data Sharing Statement

Data
Data available: No

Additional Information
Explanation for why data not available: Data generated during this study will be shared on request.
